# Feasibility and Outcomes of Endoscopic Submucosal Dissection for Colorectal Premalignant and Early-Stage Malignant Lesions at a Community Hospital Serving Rural Population

**DOI:** 10.1016/j.gastha.2026.100974

**Published:** 2026-04-20

**Authors:** Siddharth B. Javia, Whitney Reid, Seth Crockett, Gregory A. Coté

**Affiliations:** 1 Department of Gastroenterology, Confluence Health, Wenatchee, Washington; 2 Division of Gastroenterology & Hepatology, Oregon Health & Science University, Portland, Oregon; 3 Department of Pathology, Confluence Health, Wenatchee, Washington

**Keywords:** Endoscopic Submucosal Dissection, Large Colon Polyps, Community Hospital, Rural Population, Colon Cancer

## Abstract

**Background and Aims:**

Shortage of specialists and distance to facility are known barriers associated with reduced colorectal cancer screening in rural America. Although data are sparse, rural patients are at risk for undergoing colectomy for a nonmalignant polyp due to lack of local expertise with advanced endoscopic resection techniques. We aim to study the feasibility and clinical outcomes of an endoscopic submucosal dissection (ESD) at a rural US health system.

**Methods:**

ESD was introduced in a planned manner at our community hospital that predominantly serves a rural population. Procedure data and clinical outcomes were reviewed for consecutive patients who underwent attempted ESD for colorectal premalignant and early-stage malignant lesions from February 2021 to January 2024.

**Results:**

Of 92 attempted ESDs, standard ESD technique was successful in 82 lesions (90.2%), while hybrid ESD-endoscopic mucosal resection technique was required for 9 lesions (9.8%) and 1 ESD was aborted. Forty-four (47.8%) lesions were in the right colon. For those who underwent standard ESD without hybrid technique, resection rates were en bloc 81 (98.7%), R0 62 (75.6%), and curative 58 (70.7%). Adverse events occurred in 4 (4.8%). There were no delayed perforations. Final pathology showed tubular or tubulovillous adenoma in 51 (62.2%), sessile serrated lesion in 2 (2.4%), high-grade dysplasia in 14 (17.1%), and adenocarcinoma in 15 (18.3%), with adenocarcinomas being staged T1 with low-risk features in 9 (11%) and ≥T1 with high-risk features in 6 (7.3%). Surveillance colonoscopy or surgical pathology was available in 44 (53.7%). Recurrent or residual polyp was noted in 1 (1.2%).

**Conclusion:**

ESD can be introduced at a community hospital with a high level of safety and efficacy.

## Introduction

Colorectal cancer is both more common and more lethal in rural communities.^1^^,^^2^ Shortage of specialists and distance to test facilities are some of the predominant factors leading to poor adherence with colorectal cancer prevention and care.^3^^,^^4^ Surgery for nonmalignant polyps is associated with significant morbidity and mortality.^5^ With recent advances in endoscopic resection techniques, majority of these lesions can be managed with endoscopic resections appropriately, and surgery should be avoided.^6^ Endoscopic submucosal dissection (ESD) for colorectal polyps has been associated with high en bloc removal rate, low recurrence rate, and improved histopathology specimen assessment when compared to endoscopic mucosal resection (EMR).^7^, ^8^, ^9^ Availability of ESD technique for colon polyps remains limited in United States and usually only available at select academic tertiary medical centers located in population-dense areas of the country.^10^ This is predominantly due to lack of widespread expertise, few structured training programs, lack of reimbursement codes, longer procedure times, and higher adverse event rates.^10^ EMR still remains a primary technique for removal of large colorectal polyps in the United States. Piecemeal EMR is associated with recurrence rates of up to 20%, leading to frequent surveillance or therapeutic colonoscopies to evaluate and treat for any possible recurrences.^8^^,^^11^ The vast majority of patients who are discovered to have a superficial colorectal cancer after a piecemeal EMR usually end up getting an oncologic surgical resection because margin assessment and assessment of depth of invasion is frequently limited or not possible.^12^^,^^13^ The aims of this study were to determine the feasibility and clinical outcomes of establishing a colonic ESD program within a community hospital center serving predominantly rural communities.

## Methods

This study was conducted at a single regional health system, Confluence Health Hospital-Central Campus. This is the largest medical center in the North Central Washington region that serves a predominantly (approximately 75%) rural patient population of 250,000 individuals across a 150-mile radius. The nearest academic tertiary hospital center which offered ESD procedure was 150 miles away from our hospital location. The gastroenterology practice includes 7 gastroenterologists, one of who acquired skills to perform ESD. General surgery was available for surgical back up. Colorectal surgery was not available at our hospital. Prior to the launch of this ESD program, large polyps were removed using EMR technique. Surgical resections were typically reserved for malignant polyps that did not meet the criteria for curative resection. The practice consolidated all large (≥2 cm) polyp referrals to this provider, which the other practitioners performed EMR or polypectomy of small to medium size polyps (<2 cm) or if polyp characteristics appeared higher risk. The decision to perform ESD over EMR depended on the following characteristics: (1) presence of high-risk features for superficial invasion, for example, nongranular polyp, especially pseudodepressed type, granular mixed type polyps with dominant nodule, and Japanese NBI Expert Team IIb pattern; (2) lesions over 30 mm in size without high-risk endoscopic features; and (3) willingness of patient to undergo a longer procedure with higher procedural risks but potentially improved outcomes in terms of lower recurrence rate and higher en bloc and curative resection rates.

Therefore, we reviewed the following plan to introduce this procedure at our community hospital center. The local credentialing process was established after reviewing credentialing criteria from other institutions in the state where this procedure was recently introduced. The criteria were discussed with the credentialing committee, where other specialties including surgeons were present. As a part of the credentialing process, the following conditions had to be met:

1) Proficiency in EMR technique, preferably completion of advanced endoscopy training.
2) Completion of 3 didactic courses specific for ESD.
3) Completion of 3 successful ex vivo ESD training sessions.
4) After meeting these initial criteria, first 3 rectal ESD cases were required to be performed under direct supervision of a proctor proficient in ESD technique.

**Figure 1:**
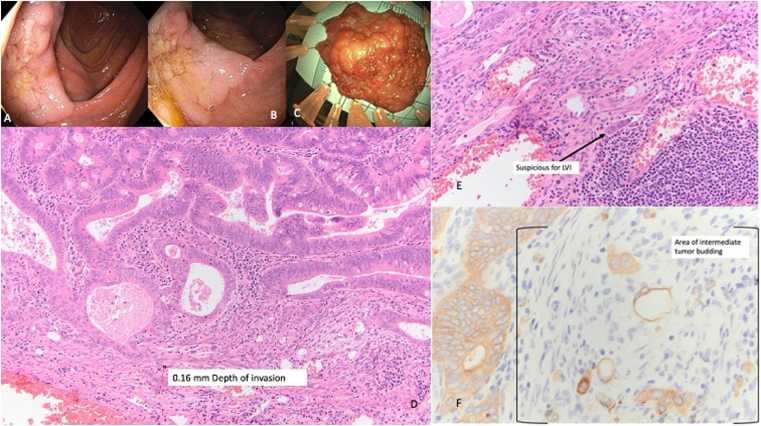
(A) Cecal polyp. (B) View of the polyp in the cecal groove. (C) Pinned specimen of the cecal polyp on a scaled foam board. (D) Depth of tumor invasion: 160 micrometers. (E) Features suspicious for lymphovascular invasion. (F) Area of intermediate tumor budding. LVI, lymphovascular invasion level 1.

After initial credentialing, the physician group decided to restrict ESD procedures to the rectum and left colon. After successful completion of 5 left colon ESDs, the application of ESD was expanded to right colonic and upper gastrointestinal tract lesions. For this study, we only include outcomes of colonic premalignant and early-stage malignant lesions. We included all the cases that were referred for and had undergone attempted ESD for removal of colonic premalignant and early-stage malignant lesions from Feb 2021 to Jan 2024. We began performing ESDs in Feb 2021, starting with 3 rectal ESD cases performed under direct supervision of a proctor. S.J. had not performed ESDs previously other than ex vivo training sessions. Total number of ESD performed by S.J. for each 3-month period was also assessed. We obtained further procedural and outcome information on lesions that underwent ESD procedure. Demographic information including distance of residence to performing location, lesion characteristics, ESD procedure, final pathology, adverse events, surveillance, and recurrences were reviewed. Technical success was defined as ESD completion using standard ESD technique or hybrid ESD-EMR technique. Standard ESD technique was defined as completion of ESD procedure using needle type or insulated tip knives only without the use of snare type devices. Hybrid ESD-EMR technique was defined as the use of knife as well as snare accessories for completion of lesion removal. Standard ESD techniques included pocket creation method or saline immersion technique. Polyp size estimation was performed by the proceduralist based on the dimension of the polyp on the pinned ESD specimen on a scaled foam board (Figure 1). Specimen size was documented based on pathology report measurements. We only had information on total procedure time, which included scope insertion, ESD time, hemoclip closure, and retrieval of specimen. We also noted total number of procedures where 2 lesions were removed with ESD technique. We did not remove more than 2 lesions per procedure using ESD technique for the following 2 main reasons: (1) to reduce total procedure time and (2) to allow proper treatment of delayed adverse events. En bloc resection was defined as removal of entire lesion in 1 piece. R0 resection was defined as removal of lesion with negative deep and lateral margins on final pathology. Curative resection was defined as R0 resection with “low risk” pathology features as per European Society of Gastrointestinal Endoscopy guidelines^14^—negative lateral and deep margins, moderate to well differentiated morphology, absence of lympho-vascular invasion, low tumor budding rate, and depth of invasion less than 1000 μm for sessile polyps. We also collected information on R1 resections due to “focally positive lateral margins” since this usually represented mucosal incision cautery artifact extending to polyp margin or tearing of lateral margin during specimen retrieval or specimen pinning; occasionally, this also happened due to traction process. Adverse events were assessed based on chart review of a hospital admission for an adverse event at the performing location or outside hospital location (patient reported). Severe delayed bleeding was defined as any bleeding that resulted in hospital admission. All lesions with adenocarcinoma were discussed at multidisciplinary tumor board for consideration of additional treatment vs surveillance. Surveillance colonoscopies were typically recommended at 1–2-year interval based on pathology. Surveillance colonoscopy interval and recurrence rate were noted. Recurrence was defined as presence of prior adenomatous or serrated lesion at the site of ESD scar. This study was approved by the institutional review board.

### Statistics

Data collection and analysis were performed using Microsoft Excel. *T*-test and Chi-square test were used for determination of *P* value. Statistical significance was defined by a *P* value of <.05.

## Results

A total of 92 ESDs (Table 1) were attempted for colorectal premalignant and early-stage malignant lesions from February 2021 to January 2024 by the provider who offered both EMR and ESD procedures. Median age was 68 (interquartile range [IQR], 60–74). Of these, 57 (62%) were males and 35 (38%) were females. The majority were American Society of Anesthesiologists (ASA) class II 58 (63%) and III 31 (34%). Median body mass index was 29.4 (IQR, 25.1–35.8). Median travel distance in miles was 23 (range 0–136 miles). Thirty-six (39.1%) lesions were removed from patients who lived in the same city (travel time considered as 0 miles), while remaining 56 (60.9%) lesions were removed from patients who lived in rural communities. Eighty-six percent of lesions were found in White patients, while 5% in Hispanic patients. Forty-four (47.8%) lesions were in the right colon and 48 (52.2%) lesions were in the left colon/rectum. Median lesion size was 30 mm (IQR, 20–40). Median specimen size was 33 mm (IQR, 26–43). Median procedure time for single lesion removal was 125 minutes (IQR, 90–161). Japanese NBI Expert Team classification was IIa, IIb, and III in 48 (52%), 43 (46.7%), and 1 (1.1), respectively. The majority of lesions (86) involved less than half of circumference, while 5 lesions involved more than 50% of circumference, and 1 lesion was circumferential.

**Table 1:** Baseline Characteristics of Patients Who Underwent Attempted ESD Procedure

Baseline characteristics n (%)	
Total patients	92
Age, median (IQR)	68 (60–74)
Sex	
Male	57 (62)
Female	35 (38)
ASA I	2 (2)
ASA II	58 (63)
ASA III	31 (34)
ASA IV	1 (1)
BMI (IQR)	29.4 (25.1–35.8)
Median travel distance in miles (IQR)	23 (0–68)
Race	
White	86 (93.5)
Hispanic	5 (5.4)
Unknown	1 (1.1)
Right colon	44 (47.8)
Left colon and rectum	48 (52.2)
Median size (IQR)	30 (20–40)
Median specimen size mm (IQR)	33 (26–43)
Median procedure time (IQR) single lesion ESD (n = 80)	125 (90–161)
Median procedure time (IQR) multiple lesion ESD (6 procedures, 12 lesions)	180 (122–244)
Japan NBI Expert Team (JNET) classification	
IIa	48 (52.2)
IIb	43 (46.7)
III	1 (1.1)
Bowel circumference	
Less than 50%	86 (93.4)
More than 50%	5 (5.4)
Circumferential	1 (1.1)
Technical success (standard ESD + hybrid ESD-EMR)	91 (98.9)
Standard ESD technique	82
Hybrid ESD-EMR technique (knife + snare removal)	9
Number of knives used	
1	80 (87)
2	12 (13)
Traction	3 (3.3)
Overtube use	0 (0)
Clip closure	53 (57.6)
Postprocedure hospital admission	3 (3.3)

ASA, American Society of Anesthesiologists; BMI, body mass index.

Eighty-seven percent of lesions were removed with 1 cautery knife, while 13% needed 2 or more for completion. Traction was used in only 3.3%. Overtube type accessories were not available for use in difficult conditions (eg, scarred polyp or looping). Saline immersion technique was frequently used for difficult conditions in 23 lesions (25%). Clip closure of resection defect was achieved in 53 (57.6%). We did not use suture closure for any of the resection defects.

**Figure 2:**
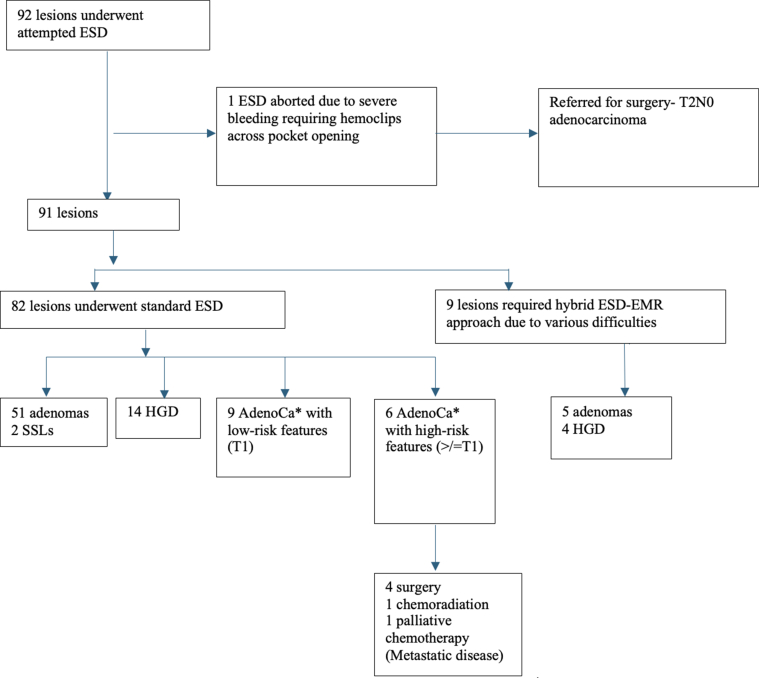
Flowchart of lesions that underwent attempted ESD. SSL, sessile serrated lesion; AdenoCa∗, adenocarcinoma; HGD, high-grade dysplasia.

Out of 92 attempted ESDs, 1 was unsuccessful due to severe bleeding from the pocket, which required hemoclip application and closure of the pocket (Figure 2). Additionally, there were findings of severe submucosal fibrosis vs deep submucosal tumor infiltration. This patient was referred for oncologic surgical resection without endoscopic resection. This patient turned out to have T2N0 adenocarcinoma on surgical pathology. Of the remaining 91 lesions, 9 lesions required use of hybrid ESD-EMR technique due to various difficulties encountered during the procedure including severe fibrosis, difficult colonoscope position or prolonged procedure time. The remaining 82 lesions were successfully removed with standard ESD technique without use of snare device. For these 82 lesions that underwent standard ESD, en bloc, R0, and curative resection rates were 81 (98.7%), 62 (75.6%), and 58 (70.7%), respectively (Table 2). Focally positive margins (as described by the pathologist) led to non-R0 resection in 12 (14.6%) lesions and noncurative resection in 10 (12.2%) lesions. Postprocedure hospital admissions were rare (2, 2.4% in standard ESD group). Adverse events were reported in 4 out of 82 (4.8%) in standard ESD group. One had severe delayed bleeding requiring hospital admission and repeat endoscopic intervention. One (1.1%) had postprocedure anal pain, which was treated conservatively and 1 patient with circumferential anorectal ESD (1.1) presented with anal canal stenosis and related bowel obstruction 3 weeks after the procedure.

**Table 2:** Comparison of Standard ESD vs Hybrid ESD-EMR Technique

	Standard ESD (n = 82)	Hybrid ESD-EMR (n = 9)
Location		
Right colon	36	8
Left colon and rectum	47	1
Size median mm (IQR)	30 (20–40)	35 (25–47.5)
En bloc resection (%)	81 (98.8)	4 (44.4)
R0 resection	62 (75.6)	3 (33.3)
Curative resection	58 (70.7)	3 (33.3)
Adverse event	4 (4.9)	2 (22.2)
Bleeding	1 (1.2)	2 (22.2)
Closure	46 (56.1)	7 (77.7)
Delayed perforation	0	0
Final pathology		
Tubular or tubulovillous adenoma	51 (62.2)	5 (55.6)
Sessile serrated lesion	2 (2.4)	0
High grade dysplasia	14 (17.1)	4 (44.4)
Adenocarcinoma	15 (18.3)	0

There were no delayed perforations or surgery for the treatment of an adverse event. Final pathology of 82 standard ESD lesions were tubular or tubulovillous adenoma in 51 (62.2%), sessile serrated lesion in 2 (2.4%), high-grade dysplasia in 14 (17.1%), T1 adenocarcinoma with low-risk features in 9 (11%), and ≥T1 adenocarcinoma with high-risk features in 6 (7.3%) (Figure 1, Supplementary Table 1).

Five patients had died by the time data collection was completed. Two died of congestive heart failure, 1 died of decompensated cirrhosis, 1 died of metastatic esophageal cancer, and 1 patient who had metastatic rectal cancer also died of cancer-related causes. Median time to death was 11 months.

**Figure 3:**
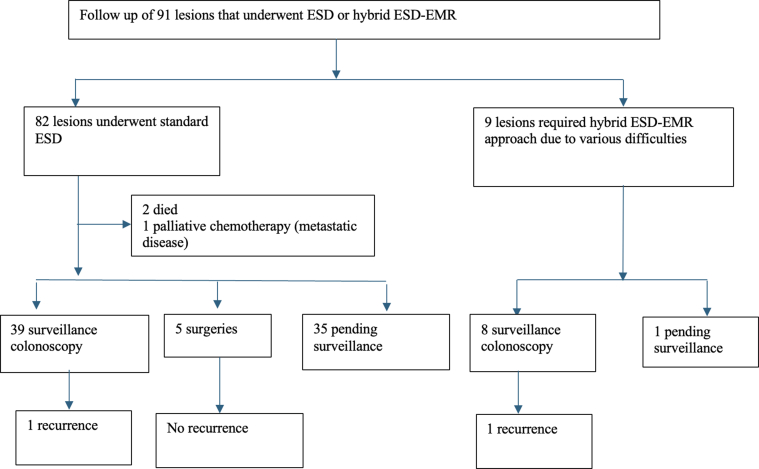
Flowchart of follow-up of 91 lesions that underwent ESD or hybrid ESD-EMR.

Surveillance data were available. Surveillance was defined by colonoscopy, surgical pathology, or both. Surveillance was available in 44 out of 82 lesions in standard ESD group (Figure 3). There was only 1 residual adenoma (2.4%), which was removed endoscopically. Thirty-five lesions were still awaiting surveillance colonoscopy (42.7%). Five out of 11 patients who had focally positive margins on initial histopathological diagnosis had surveillance colonoscopy available. None of these 5 patients had any residual or recurrent polyp at prior ESD scar location.

For those who underwent Hybrid ESD-EMR technique (n = 9), en bloc, R0, and curative resection rates were 4 (44%), 3 (33.3%), and 3 (33.3%), respectively (Table 2). Eight out of 9 hybrid ESD-EMR were performed in the right colon, while 1 was performed in the left colon/rectum. Adverse events occurred in 2 lesions in this group, both of which were severe delayed bleeding requiring hospital admission and endoscopic interventions. Final pathology of 9 lesions that underwent hybrid ESD-EMR technique were adenoma in 5 (55.5%) and high-grade dysplasia in 4 (44.4%) (Figure 2). Surveillance colonoscopy was available in 8 out of 9 in hybrid ESD-EMR group. There was 1 residual adenoma in hybrid ESD-EMR group, which was removed endoscopically.

Median surveillance colonoscopy interval was 12 months.

### Q1–Q12 Results

**Figure 4:**
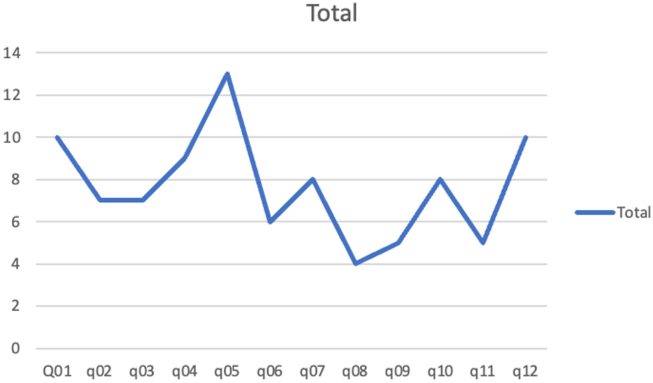
ESD numbers per quarter Q1-Q12 (February 2021 to January 2024).

Figure 4 shows total number of ESDs performed over each quarter. Our ESD numbers and median procedure time remained relatively stable over 3 years (Table 3). We performed 2, 6, and 1 hybrid ESD-EMR cases during year 1–3, respectively. Saline immersion technique was used in 1, 1, and 21 lesions during years 1, 2, and 3, respectively.

**Table 3:** Comparison of Outcomes of Lesions That Underwent Removal Using Classic ESD Technique by Each Year

	Year 1	Year 2	Year 3
Number of lesions	30	25	27
Location			
Right colon	10 (33.3)	7 (28)	18 (66.7)
Left colon and rectum	20 (66.7)	18 (72)	9 (33.3)
Size median mm (IQR)	25 (20–35)	37.5 (20–50)	37.5 (20–45)
Resected specimen size, median, mm (IQR)	37 (24.5–38.7)	41 (26.5–53)	38 (27–45)
Median procedure time for single lesion removal (minutes)	120 (83.2–153.7)	110 (77–135)	132 (97.5–156.5)
Hospital admission	2 (6.7)	0	0
En bloc resection	30 (100)	24 (96)	27 (100)
R0 resection	25 (83.3)	17 (68)	20 (74.1)
Curative resection	24 (80)	17 (68)	17 (63)
Non R0 due to focally positive margins	2 (6.7)	5 (20)	5 (18.5)
Noncurative resection due to focally positive margins	2 (6.7)	3 (12)	5 (18.5)
Adverse event	2 (6.7)	1 (4)	1 (2.7)
Surveillance colonoscopy or surgery	21 (70)	18 (72)	5 (18.5)
Residual or recurrent adenoma	1 (3.3)	0	0
Awaiting surveillance^a^	8 (26.7)	5 (20)	22 (81.5)

a Excluding those who died or had metastatic disease.

## Discussion

In this single-center retrospective study, we report outcomes of ESD performed for colorectal premalignant and early-stage malignant lesions over 3-year period at a community hospital that predominantly serves rural communities. We carried out careful implementation of ESD at our center after developing a credentialing process that was acceptable to credentialing committee and gastroenterology department. Our goal was to provide this procedure locally for our rural communities with a high level of safety and efficacy.

Our en bloc resection rates were high but R0 and curative resection rates were just below the benchmark of 80% and 75%, respectively, as per European Society of Gastrointestinal Endoscopy consensus guideline; however, the guideline notes that this goal may be unrealistic, even for expert endoscopists.^14^ A multicenter study from North America by Draganov et al found that en bloc, R0, and curative resection rates for colon lesions were 85.8%, 83.4%, and 83.9%, respectively, while rates for rectal lesions were 88.8%, 85.6%, and 79.8%, respectively. They also determined that colorectal lesions had significantly higher likelihood of failed en bloc resection.^15^ Our en bloc resection rates for colorectal lesions were comparable to this study, but R0 and curative resection rates were marginally lower. In our cohort, marginally lower R0 and curative resection rate were attributed by “focally” positive lateral margins, which reduced R0 and curative resection rates by 14.6% and 12.2%, respectively. Surveillance colonoscopy was available in 5 out of 11 lesions that had “focally” positive lateral margins, none of these patients had recurrent or residual polyp at the site of prior ESD. There were only 2 recurrences noted in the entire cohort. Small recurrence was noted in 1 patient who had undergone standard ESD for a large 8 cm anorectal polyp with over 50% circumference at anal canal, and the other patient had undergone hybrid ESD-EMR. Both recurrences were small and easily treated. This is one of the main merits of ESD that by achieving a higher en bloc resection and lower recurrence rates, we could possibly reduce total number of colonoscopies required for surveillance after a piecemeal EMR.

En bloc, R0, and curative resection rates for hybrid ESD-EMR technique remained significantly lower compared to standard technique, which is in line with previously published studies.^16^ Lesions that had undergone hybrid ESD-EMR were exceptionally difficult cases, many of them had significant scarring from prior resections or injectate-related granuloma underneath a scarred polyp. We did not record presence of fibrosis from prior biopsy, polypectomy, or injectate-related granuloma reaction. But we believe that our data included all of these situations and represented real-world scenario where polyps with submucosal fibrosis are frequently referred for ESD and this can make ESD procedure challenging. Due to the use of hybrid technique in difficult cases only rather than a primary treatment method and due to low numbers in hybrid ESD-EMR group (n = 9), we do not think we can arrive to any meaningful conclusions in the hybrid ESD-EMR group. Despite having had over two-thirds of the lesions in the right colon during year 3, we performed hybrid ESD-EMR in 1 lesion only. This likely reflects improvement in our ESD technique, which included increasing use of the saline immersion technique. We performed the saline immersion technique in 75% of the lesions during year 3. We did not have the overtube type accessory available at our center due to limited resources. Although approximately half of the lesions were in the right colon (47.8%), we were able to carry out ESD successfully with use of saline immersion technique in many of these lesions, which allowed completion of ESD without use of overtube type accessories. We find that saline immersion technique is a very useful technique for ESD in the right colon where ESD could become challenging due to looping or overdistention of the colon, especially with prolonged procedure time.^17^

Our ESD volume remained similar over the study period of 3 years. This is likely related to limitations of referral population in rural setting and lack of formal marketing. We did not include esophageal or gastric ESDs in this study as it was aimed at looking at outcomes of ESD regarding colon cancer prevention and treatment.

The median distance traveled to get this procedure was 28 miles. Patients traveled from rural communities; the route can often require travel through a mountainous region, making winter travel difficult. This is the only study to our knowledge which evaluates the feasibility of establishing an ESD program and reporting outcomes of ESD at a community hospital that predominantly serves rural patients. This study demonstrates that ESD can be introduced and offered effectively and safely with proper planning. This is particularly important in a rural setting where there are higher rates of surgery for premalignant colon polyps.

As per a recent study from the United States, as many as 25% of colon surgeries that are performed for colon polyps and cancers are performed on nonmalignant polyps.^6^ We believe that many of our patients were able to avoid a surgery due to availability of ESD technique locally.

This study is limited by its single-center, single-provider design. Adverse events were not measured systematically but rather by patient self-report. The reported AE rate is likely accurate as it is unlikely that patients would have developed a serious AE without presenting at the treating facility given its remote location.

## Conclusion

ESD services are feasible in a rural community with outcomes that approximate international guidelines. Longer-term studies involving multiple health systems will be needed to confirm the incremental value of ESD over EMR for advanced colorectal polyps and whether outcomes are comparable to the highest volume referral centers typically located in more population-dense communities. Such studies should involve patient advocates to measure the impact of local expertise on patient-centered outcomes.

## Supplementary Materials

Supplementary Table 1

Extended PDF
