# Supplementary material for: Feasibility and Outcomes of Endoscopic Submucosal Dissection for Colorectal Premalignant and Early-Stage Malignant Lesions at a Community Hospital Serving Rural Population
Source: Gastro Hep Adv. 2026 Apr 20;5(7):100974. doi: 10.1016/j.gastha.2026.100974 (PMC13217485; doi:10.1016/j.gastha.2026.100974)
Supplement: Supplementary Table 1 [file mmc1.pdf]

Supplementary table 1: >/=T1 adenocarcinoma with high-risk features. Sessile polyps=Lymphovascular invasion (LVI), poor differentiation, >1000 micrometer invasion, intermediate to high tumor budding (TB) rate

	Location	size	Morphology	En bloc	R0	Pathology features	Definitive therapy	Recurrence or residual lesion on surgical pathology or surveillance colonoscopy
1	IC valve	50	IIa+Is	yes	yes	<1000 micrometer invasion, Intermediate TB score, LVI suspicion	surgery	no
2	IC valve	15	IIa	yes	no	2000 micrometer invasion	surgery	no
3	Anorectum	20	Is	yes	no	positive deep and lateral margins, metastatic lymphadenopathy	Palliative chemotherapy	-
4	Ascending colon	40	IIa	yes	yes	300 micrometer invasions, LVI	Surgery	no
5	Rectum	20	IIa	yes	yes	2500 micrometer invasion, LVI, intermediate TB score	Chemoradiation	no
6	Ascending colon	35	Is	yes	yes	<1000 micrometer, LVI suspicion, intermediate TB score	surgery	no
